# Protective effect of aplysin on liver tissue and the gut microbiota in alcohol-fed rats

**DOI:** 10.1371/journal.pone.0178684

**Published:** 2017-06-16

**Authors:** Meilan Xue, Ying Liu, Rui Lyu, Na Ge, Man Liu, Yan Ma, Hui Liang

**Affiliations:** 1 Qingdao University of Medicine, Qingdao, PR China; 2 Baotou Medical College, Inner Mongolia University of Science and Technology, Baotou, PR China; University of Louisville School of Medicine, UNITED STATES

## Abstract

**Background:**

This study investigated the protective effect of aplysin on the liver and its influence on inflammation and the gut microbiota in rats with ethanol-induced liver injury.

**Methods:**

Male Sprague-Dawley rats were randomly assigned to an alcohol-containing liquid diet, control liquid diet or treatment with aplysin for 8 weeks. Hepatic and intestinal histopathological analysis was performed, and cytokine levels and the intestinal mucosal barrier were assessed. Enterobacterial repetitive intergenic consensus polymerase chain reaction (ERIC-PCR) and 16S rDNA high-throughput sequencing were performed to provide an overview of the gut microbiota composition.

**Results:**

Chronic alcohol exposure caused liver damage in rats. Serum aspartate aminotransferase (AST), aminotransferase (ALT), alkaline phosphatase (ALP) and triglyceride (TG) activities in liver tissue were higher than in the control group. Alcohol administration elevated the levels of serum transforming growth factor-β (TGF-β) and tumor necrosis factor-α (TNF-α) and reduced interleukin-10 (IL-10) levels compared with those of control rats. In addition, the levels of plasma endotoxin, diamine oxidase (DAO), and fatty acid-binding protein 2 (FABP2) in the alcohol group were higher than in the control group. The results of ERIC-PCR indicated that aplysin treatment shifted the overall structure of the ethanol-disrupted gut microbiota toward that of the control group. One hundred twenty to 190 genera of bacteria were detected by high throughput sequencing. Alcohol-induced changes in the gut microbial composition were detected at the genus level. These alcohol-induced effects could be reversed with aplysin treatment.

**Conclusions:**

These results suggest that aplysin exerts a protective effect on ethanol-induced hepatic injury in rats by normalizing fecal microbiota composition and repairing intestinal barrier function.

## Introduction

Chronic excessive ethanol consumption leads to hepatic damage, which may result in the development of alcohol-induced liver disease (ALD). ALD is a serious health problem among alcoholics and continues to be a major health issue worldwide. ALD is characterized by the development of fatty liver, steatohepatitis, inflammation, hepatocellular necrosis and hepatocellular carcinoma [1–3].

Studies have shown that alcohol intake leads to distinct change in intestinal microbiota communities and dysbiosis in animals and humans [4–6]. The intestinal microbiota contributes to an individual’s susceptibility to alcoholic liver disease [7]. Alcohol ingestion can impair intestinal barrier defense and induce physical disruption of the intestine [8, 9]. In the small intestine, alcohol and its metabolites encounter a physical barrier lining the intestinal tract, which is important in maintaining barrier integrity and preventing invasive pathogens from moving into the body. Alcohol may also cause loss of gut barrier function by extracting and dissolving lipids from the mucus with a resultant decrease in mucosal surface hydrophobicity, which is a critical component of gut barrier function [10].

Alcohol intake could interfere with the immune system in the small intestine, which can distinguish between symbiotic bacteria that normally live in the human gut and foreign microorganisms that cause disease. Long-term alcohol intake may result in the leakage of microbes and microbial products from the gut tract, which can quickly reach the liver through the portal system. They can dysregulate the immune signaling pathway in the liver and stimulate the production of proinflammatory cytokines, including tumor necrosis factor-α (TNF-α), interleukin-8 (IL-8) and IL-1β, which reduce cellular dysfunction and aggravate liver damage [11].

Furthermore, the intestinal mucus or epithelial barrier becomes destroyed, which can induce dysbiosis and pathogenic bacterial translocation. The dysbiosis in the intestine increases intestinal permeability [12]. Therefore, various gut-derived bacterial products, including bacterial endotoxins, will reach the liver directly via the portal vain system and contribute to ALD [13]. Endotoxins are lipopolysaccharides (LPSs) derived from cell walls of gram-negative bacteria. Endotoxemia was found in ALD patients, and gut leakage appears to be the cause of endotoxemia in ALD [14]. Therefore, the modulation of intestinal microbial structure and restoration of the intestinal mucous barrier will be helpful for the prevention and treatment of alcoholic liver injury.

Aplysin, molecular formula C15H19OBr, is a brominated sesquiterpene from the red alga Laurencia tristicha, with a molecular weight of 295, as previously described [15]. Aplysin has attracted much attention because of its potent pharmacological activities, including antitumor and antioxidant activities [16–18]. Our previous studies show that aplysin can reduce ethanol-induced hepatic injury in rats through attenuating oxidative stress, modulating ethanol metabolizing enzyme activity, and inhibiting mitochondrial damage-mediated apoptosis [15]. However, its potential effect on the gut microbiota and inflammation in rats with ethanol-induced liver injury has not been explored yet.

Therefore, the goal of this study was to assess the influence of aplysin on the intestinal mucosal barrier, gut microbiota and inflammation in rats with ethanol-induced hepatic injury. The present study provides a complete profile of alcohol-induced alterations in the microbiota and investigates the modulating effect of aplysin on the gut microbiota at the genus level by 16 s rDNA high-throughput sequencing.

## Materials and methods

### Ethics statement and animals

All experimental procedures were performed according to the National Institutes of Health Guide for Care and Use of Laboratory Animals (Publication No. 85–23, revised 1985). The protocol was approved by the Review Committee for the Use of Human or Animal Subjects of Medical College of Qingdao University. All efforts were exerted to reduce the suffering of experimental animals.

Adult male Sprague-Dawley (SD) rats weighing 180–220 g were purchased from the Experimental Animal and Animal Experiment Center (Qingdao China). Rats were maintained in our animal facility at a set temperature (22–25°C) and humidity (50%-60%) and were on a 12 h light-dark cycle. Animals were allowed one week for acclimatization before experimentation and were allowed free access to rodent chow and tap water throughout the study.

### Chemicals

Aplysin (molecular formula C15H19OBr, M.W. 295) was separated and purified by our laboratory from the red alga L. tristicha. The purity of aplysin was 97.6% as determined by the Institute of Oceanology, Chinese Academy of Sciences. Aplysin was extracted from dried plant material following previous procedures [19, 20]. Aplysin is a type of liposoluble substance that is soluble in ethanol or other organic solvents. The rest of the chemicals applied in this study were of an analytical grade.

### Experimental design and treatment protocol

Forty-five male SD rats were randomly divided into the following three groups (15 rats in each group): C, control group, normal diet gavaged with normal saline; M, ethanol-treated model group, normal diet gavaged with ethanol (56% (v/v) ethanol 8 mL•kg^-1^ •d^-1^ 2wk + 12 mL•kg^-1^ •d^-1^ 6 wk); A, aplysin treatment group, normal diet gavaged with aplysin and ethanol (aplysin 150 mg•kg^-1^ •d^-1^, ethanol dose was same as model group). Two hours after alcohol or saline lavage, rats of control and model groups were supplemented orally with 1 mL soya bean salad oil via an intragastric tube for 8 weeks. Rats in the aplysin treatment group were supplemented orally with 150 mg•kg-1 •d-1 of aplysin (dissolved in soya bean salad oil)via an intragastric tube for 8 weeks.

The type of alcohol is Red Star Erguotou [56% (v/v) ethanol] purchased from Beijing red star co., LTD.

The normal diets were purchased from the Experimental Animal and Animal Experiment Center (Qingdao, China). These diets contained carbohydrates, protein, lipids, vitamins, minerals and so on (summarized in Table 1).

**Table 1:** Composition of the diets used in this study.

The composition of the diets	(g/Kg)
Casein	200.0
DL-Methionine	3.0
Sucrose	500.0
Corn starch	150.0
Corn oil	50.0
Cellulose	50.0
Mineral mix, AIN-76 (170915)	35.0
Vitamin mix, AIN-76A (40077)	10.0
Choline bitartrate	2.0
Ethoxyquin, antioxidant	0.01

The experiment lasted 8 weeks. Stool samples were collected using metabolic cages after 12 h from the last lavage. The rats were euthanized by intraperitoneal injection of 7% chloral hydrate at the end of 8 weeks. The blood was collected by an aortic abdominalis puncture and centrifuged. The serum and blood plasma were harvested and stored in the refrigerator at -80°C. Liver and small intestine tissues were excised.

### Pathological observation of liver and intestinal tissue

After the animals were sacrificed, liver and small intestine tissues were quickly excised, rinsed with cold isotonic saline and then fixed with 10% neutral formalin and embedded in paraffin. Embedded tissues (0.9×0.9×0.5 cm) were cut by an RM 2135 rotary Microtome (LEICA, Germany) and underwent hematoxylin and eosin (HE) staining. Then, morphological changes of sections were examined using a light microscope (Olympus BX 60, Japan), and hepatic histopathological grading was assessed. Liver pathology was scored as described by Yin et al. [21]. One of the experimenters and a rodent pathology expert examined the pathological changes of the liver and intestinal tissue in a double-blind manner.

The ultrastructure of the liver and intestinal tissue was observed using a transmission electron microscope. Hepatic or intestinal tissues were cut into 1 mm^3^ sections and fixed in 3% glutaraldehyde at 4°C for 4 h. Then, all tissue sections were washed with 0.1 M phosphate buffer saline (PBS, pH 7.2), fixed with 1% osmium tetroxide at 4°C for 1 h, washed with PBS (pH 7.4) three times, dehydrated in a gradient series (30%-100%) of acetone, and then embedded with ethoxyline resin. The ultrathin sections (50–70 nm) were cut with an LKB-5 ultramicrotome (LKB, Stockholm, Sweden) and stained with 3% uranyl acetate and lead citrate. Finally, the ultrastructure of each section was examined using a JEM-1200EX transmission electron microscope (JEOL, Tokyo, Japan).

### Determination of serum aminotransferase and liver tissue TG

The activities of serum alanine amino transferase (ALT), aspartate aminotransferase (AST), alkaline phosphatase (ALP) and triglyceride (TG) in liver tissue were colorimetrically measured with commercial kits (Nanjing Jiancheng Bioengineering Corp, Nanjing, China) according to the manufacturer’s protocols.

### Plasma inflammatory factors, endotoxin and intestinal mucosal barrier assay

ELISA assay kits were applied to detect the levels of transforming growth factor-β (TGF-β), TNF-α, IL-6, IL-10 and IL-1β according to the manufacturer’s protocol (Cloud-Clone Corp, USA). All incubation steps were performed at room temperature. The optical density (OD) was read at 450 nm for all cytokines using an Elx 808 microplate reader (Biotek, Winooski, VT, USA).

The level of endotoxin in the plasma was detected by a chromogenic end-point Tachypleus amebocyte lysate (CE TAL) assay kit (Limulus Reagent Rlant Corp, Xiamen, China). The blood was collected in sterile, endotoxin-free tubes. All containers had pyrogen removed by incubating at 180°C for 24 h. The experiment was conducted in accordance with the manufacturer's instructions. Finally, the OD was read at 405 nm. The level of endotoxin was reported in endotoxin units (EU) per milliliter for plasma.

The levels of plasma diamine oxidase (DAO), D-lactic acid (D-LA) and fatty acid-binding protein 2 (FABP2) were determined by ELISA assay kits (Cloud-Clone Corp, USA) according to the manufacturer’s instructions.

### ERIC-PCR fingerprint analysis

Enterobacterial repetitive intergenic consensus (ERIC) polymerase chain reaction (PCR) analysis was used to analyze the microbial community structure of rats (14). DNA was extracted from each fecal sample with a bacterial genomic DNA extraction kit (QIAgen, Germany). PCR amplification was performed on extracted DNA as described by Khosravi et al. [22] using the primers ERIC1 (5’-ATGTAAGCTCCTGGGGATTCAC-3’) and ERIC2 (5’-AAGTAAGTACTGGGGTGAGCG-3’) in a PCR amplification instrument (Bio-Rad, USA). The reactions were performed in a final volume of 25 μL containing 2 μL of template DNA, 0.5 mL of each ERIC primer, 0.2 μL of 5 U/μL Taq DNA polymerase, 2 μL of 2.5 mmol dNTP, 2.5 μL of 10 × buffer and 17.3 μL nuclease-free water. The amplification parameters were initial denaturation at 94°C for 7 min, 30 cycles of PCR including denaturation at 94°C for 1 min, annealing at 47.3°C for 1 min, extension at 65°C for 8 min and a final extension at 65°C for 16 min. These ERIC-PCR products were separated via electrophoresis in 1% (w/v) agarose gel, stained with 0.5 μg/μL ethidium bromide (Qiagen, Germany) and analyzed under UV light in a gel documentation system (UVP, USA).

### 16S rDNA high throughput sequencing

To further examine the detailed composition of the fecal microbiome, 16S rRNA gene sequencing was performed for 15 fecal samples collected from five animas in each group after the 8 week intervention experiment; 16S rDNA high throughput sequencing was used for the analysis of the molecular ecology of fecal flora and entrusted to the Shanghai Sangon Biotechnology Corp.

Total genomic DNA was extracted with a QIAamp DNA Stool Mini Kit (QIAGEN, Germany). DNA concentration and purity were tested with a Qubit 2.0 DNA test kit (Life Technologies, USA) and 0.8% agarose gel electrophoresis. In PCR amplification, general primers (341F, 805R) targeting bacterial 16S rRNA V3 and V4 regions were used for the Miseq sequencing platform (Illumina Miseq 2 × 300, USA). After quantification and quality control, PCR products were gradually diluted and quantified. Then, 16S rDNA PCR products were sequenced using 300 bp paired-end model with the MiSeq system. The lengths of short reads were extended by finding the overlap between paired-end reads using FLASH software [23].

The raw sequence reads of fecal flora have been registered to the database: Sequence Read Archive (SRA) of GenBank of NCBI. The accession number of SRA is SRP103630.

The sequence tag barcode was used to distinguish the sample sequence. Reads were sorted according to barcode sequences and sample sources. The number of reads for each sample was counted. Quality control was conducted for each sample sequence, and the non-target area sequence, low quality data and chimeras were removed using Prinseq and Qiime software [24]. Sequences were clustered to operational taxonomic units (OTUs) using Uclust software. The operating taxon was considered a species with 0.97 domain values of sequence similarity, while the operating taxon with domain values of 0.99 was considered a genus.

Alpha diversity analysis was applied to analyze species diversity in a single sample by the evaluation of species richness (Chao), species coverage (Coverage), abundance-based coverage estimators (ACE), and species diversity (Simpson's diversity index, Shannon Index) using Mothur software [25]. The richness index Chao was used to assess the number of operational taxonomic units (OTUs) in bacterial communities. Simpson and Shannon indexes were applied to determine the diversity of the OTUs. Community structure analyses were based on phylum and genus taxonomy levels. Taxonomy-based analyses were performed through the classification of each sequence using the Ribosomal Database Project (RDP) classifier [26]. Based on Bergey’s taxonomy, the RDP classifier implied the Naive Bayesian algorithm to calculate the probability value of each sequence in the genus level. The average probability value was greater than 0.8, which was the threshold of RDP classification. Bergey's taxonomy was divided into six grades: domain, phylum, class, order, family and genus.

### Statistical analysis

The data are presented as the mean ± SD, and statistical comparisons were performed using a one-way ANOVA with SPSS software 16.0. Correlations between gut microbial composition and serological indexes were identified using bivariate correlations (SPSS 16.0). Differences were considered statistically significant when *P*<0.05.

## Results

### Effects of aplysin on histopathological changes in the liver of alcohol-fed rats

**Fig 1:**
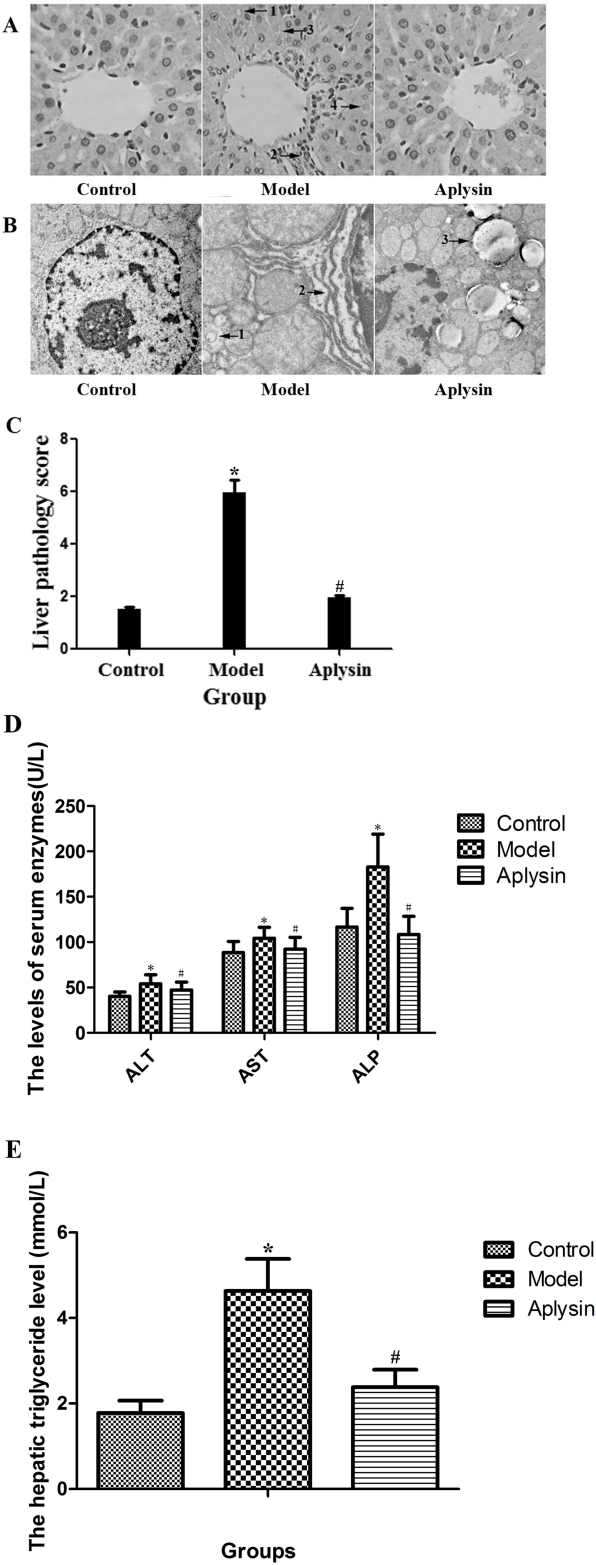
Liver histopathology and liver function changes in rats. A) Liver histopathology in rats with ethanol-induced histological changes (Magnification, ×400). The control group showed structural intactness and normal architecture. The ethanol model group showed lipid droplet accumulation (arrow 1), inflammatory cell infiltration (arrow 2), microvesicular steatosis (arrow 3) and Mallory body (arrow 4). The aplysin intervention group showed almost normal architecture. B) Hepatic ultrastructure in transmission electron microscopy (Magnification, ×20000). Hepatocytes in the control group were in good condition with little droplet fat. However, in the ethanol model group, hepatic cells showed irregular shape with increased droplet fat; the nuclear membrane was irregular or fuzzy; the mitochondria became swollen and deformed; lysosomes increased; and the ridge structure was fuzzy. In the aplysin intervention group, the morphology of hepatic cells was normal. Arrow 1: Lysosome; arrow 2: endoplasmic reticulum; arrow 3: Lipid droplet. C) The effect of aplysin on the liver pathology score in rats. The liver pathological score in the alcohol model group was significantly increased compared with that of control group rats. The liver pathology score in aplysin intervention group rats was better than that of the alcohol model group. D) The levels of serum ALT, AST and ALP. ELISA data showed that the levels of serum ALT, AST and ALP in the ethanol model group were significantly increased compared with those of control rats, whereas the aplysin intervention decreased the levels of ALT, AST and ALP. E) The hepatic triglyceride level. The TG level of liver tissue in ethanol model group was increased compared with that of control rats, whereas the aplysin intervention decreased the level of hepatic TG. *P< 0.05 vs Control; ^#^*P*< 0.05 vs Model.

Histopathological changes of the liver were assessed by light microscopy of tissue sections with H&E staining. As shown in Fig 1A, the lobular architecture in control group rats was normal. The liver cell line was radial from the center with the lobular central vein, and there was no steatosis. Alcohol exposure caused liver injury, as indicated by lipid droplet accumulation (arrow 1), inflammatory cell infiltration (arrow 2), microvesicular steatosis (arrow 3) and Mallory body (arrow 4). Liver cells in alcohol model group rats were swollen, and their pathological score significantly increased compared with that of control group rats (*P*< 0.05) (Fig 1C). These histopathological changes were alleviated by an aplysin intervention. In this group, hepatic cords arranged more regularly, and organizational structures tended to be normal. The liver pathology score in the aplysin intervention group was significantly decreased compared with that of the alcohol model group (*P*< 0.05).

Transmission electron microscopy (TEM) was used to observe the hepatic ultrastructure in different groups. As shown in Fig 1B, the hepatocytes in the control group were in good condition nearly without droplet fat. The nuclei were round or oval; the nuclear membrane was intact; the nucleolus was clear; the mitochondrial morphology was normal; the ridge structure was clear; the structure of rough endoplasmic reticulum was normal; and ribosomes were abundant. However, in the ethanol model group, hepatic cells showed an irregular shape with increasing droplet fat; the nuclear membrane was irregular or fuzzy; mitochondria started swelling and became deformed; lysosomes increased; and ridge structure was fuzzy. The rough endoplasmic reticulum was swollen, fractured and disorganized. In the aplysin intervention group, the morphology of hepatic cells tended to be normal. The nuclear membrane was intact; droplet fat in the cytoplasm decreased; and the mitochondrial structure was nearly normal. Furthermore, pathological changes were significantly reduced. The degradation of the rough endoplasmic reticulum and its disordered arrangement was improved.

### Effects of aplysin on enzymology indexes of liver function and TG

ELISA data showed that the levels of serum ALT, AST and ALP in the ethanol model group were significantly increased by 33.9%, 18.0% and 56.5%, respectively, compared with those of control rats (*P*< 0.05, Fig 1D). Moreover, the level of triglyceride (TG) in liver tissue was increased (*P*< 0.05, Fig 1E). Aplysin intervention decreased the levels of ALT, AST and ALP by 13.0%, 11.5% and 41%, respectively, compared with those of model rats (*P*< 0.05). The level of TG in liver tissue was also decreased.

### Pathological changes of small intestine tissue in light microscopy and electron microscopy

**Fig 2:**
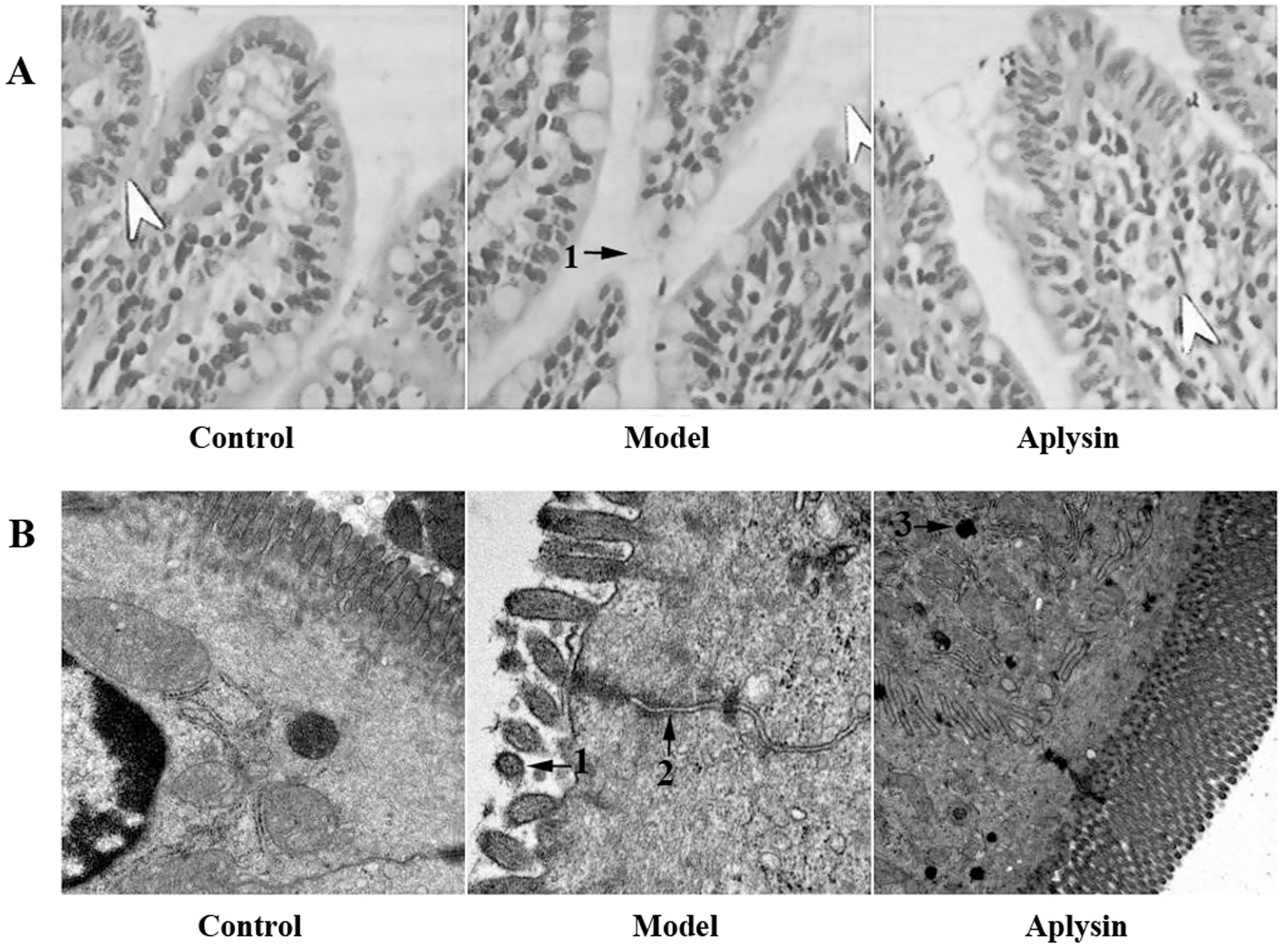
Small intestine pathological changes in rats. A) Effect of aplysin on small intestine pathological changes (Magnification, × 400). The histological features shown after HE staining indicated that the small intestinal structure is normal, and intestinal villi are lined in neat rows in the control group. However, in the alcohol model group, small intestinal villi showed obvious atrophy. There was no obvious abnormity in the aplysin intervention group. 1: Atrophy of a small intestinal villus. B) Effect of aplysin on the ultrastructure of the small intestine of rats (Magnification, × 20000). In transmission electron microscopy, small intestine microvilli in the alcohol model group were arranged sparsely, and tight connections were enlarged. After the aplysin intervention, the small intestine columnar epithelium and microvilli arrangement were improved to varying degrees, and the swelling of tight connections was reduced. 1: the broadened cell connection; 2: sparse structure of small intestine microvilli; 3: lysosomal.

The histological features shown in Fig 2A after HE staining indicate that the small intestinal structure is normal, and intestinal villi were lined in neat rows in the control group. However, in the alcohol model group, small intestinal villi structures showed obvious atrophy. There was no obvious abnormity in the aplysin intervention group.

In transmission electron microscopy, small intestinal epithelium columnar cells were in alignment, small intestinal villi were rich, and the close connection between cells was complete and clear in the control group (Fig 2B). Small intestine microvilli in the alcohol model group were sparsely arranged, and the tight connections were enlarged. After the aplysin intervention, the small intestine columnar epithelium and microvilli arrangement was improved to varying degrees, and the swelling of tight connections was reduced.

### Effect of aplysin on plasma endotoxin level and intestinal mucosal barrier function

DAO is an intracellular enzyme in the intestinal mucosa that can catalyze the oxidation of diamines, including cadaverine, putrescine and histamine. The majority of DAO activity in the blood comes from the intestine. The plasma DAO level is a useful indicator of the severity of mucosal injury and a sensitive marker of intestinal permeability [27, 28]. D-LA is a metabolic product of bacteria present in the intestinal lumen. The activity of the mucosal enzyme D-LA is also a reliable marker of intestinal mucosal integrity [29]. FABP-2, also named as intestinal fatty acid binding protein, is specifically expressed in intestinal epithelial absorbing cells and is released into the blood once the intestinal mucosa is damaged [30].

**Fig 3:**
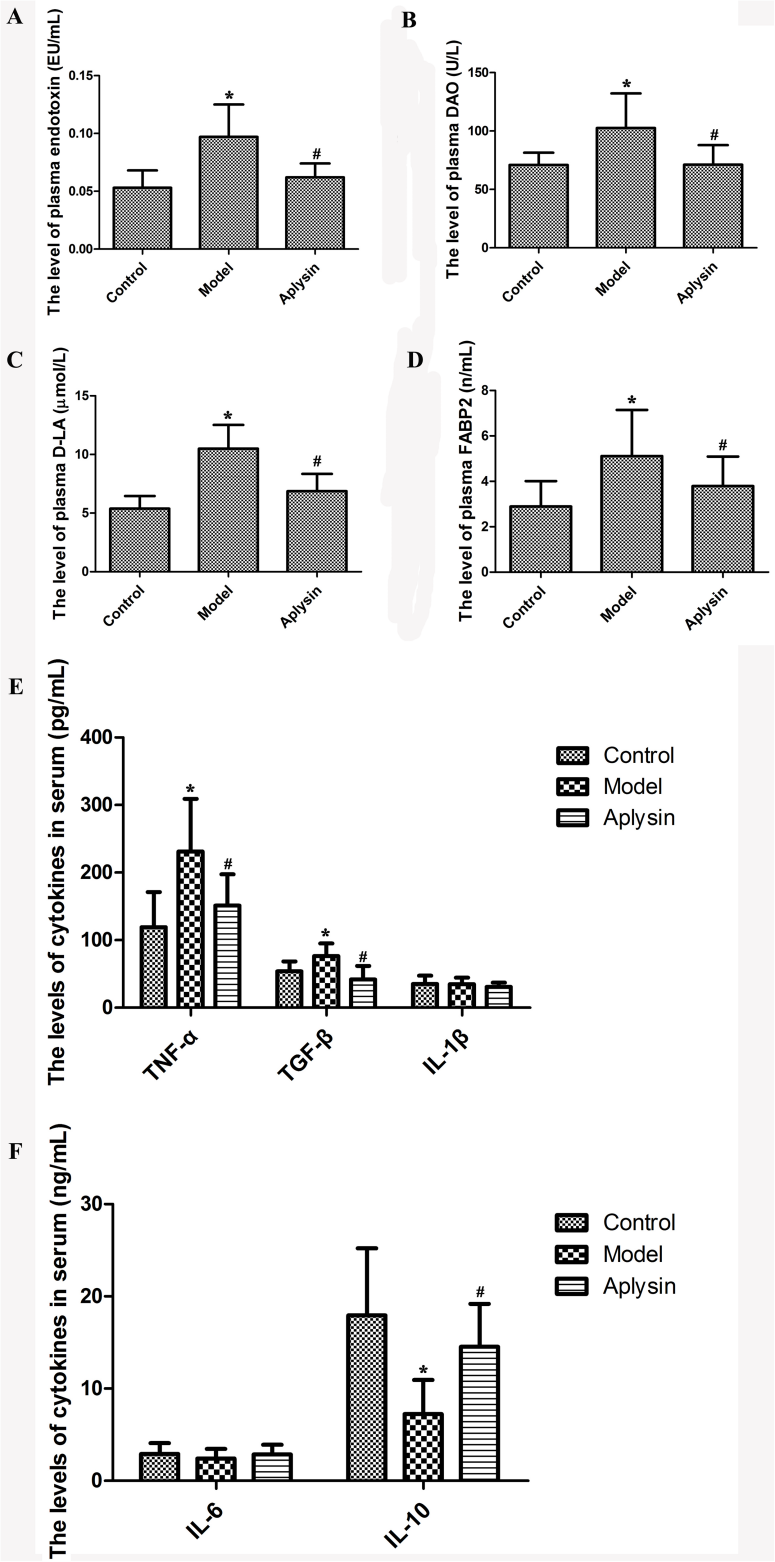
Effect of aplysin on plasma endotoxin levels, intestinal mucosal barrier function and cytokines. A) The level of plasma endotoxin in rats. B) The level of plasma DAO in rats. C) The level of plasma D-LA in rats. D) The level of plasma FABP2 in rats. E) The levels of serum TNF-α, TGF-β and IL-1β in rats. F) The levels of serum IL-6 and IL-10 in rats. The levels of plasma endotoxin, DAO, D-LA and FABP2 in the alcohol model group were higher than those in the control group. After aplysin intervention, endotoxin, DAO, D-LA and FABP2 levels were reduced compared with those of the alcohol model group. Alcohol administration elevated the levels of serum TNF-α and TGF-β and reduced the level of IL-10 compared with those of control rats. These alcohol-induced effects could be reversed with aplysin treatment. IL-1β and TNF-α levels in the aplysin group were decreased, and the level of IL-10 was increased. *P< 0.05 vs Control; ^#^*P*< 0.05 vs Model.

As shown in Fig 3, the levels of plasma endotoxin, DAO, D-LA and FABP2 in the alcohol model group were increased compared to those in the control group (*P*< 0.05). After aplysin intervention, endotoxin, DAO and FABP2 levels were reduced by 6.94%, 13.72% and 21.41%, respectively, compared with those of the alcohol model group (*P*< 0.05). The level of plasma D-LA was also decreased compared to the alcohol model group (*P* < 0.05).

### Effect of aplysin on inflammation and IL-1β, IL-6, IL-10, TGF-β and TNF-α levels

The levels of serum TNF-α and TGF-β in the ethanol model group were elevated and reached 1.9 and 1.2 times those in the control group, respectively. However, alcohol administration reduced IL-10 levels by 59.7% compared with that of control rats (all *P*< 0.05, Fig 3). These alcohol induced effects were reversed with aplysin treatment. IL-1β and TNF-α levels in the aplysin group were decreased by 34.7% and 37.0%, respectively. In the alcohol model group, the level of IL-10 was increased to 2 times than that in the alcohol model group (*P*< 0.05).

### ERIC-PCR fingerprint analysis of the gut microbiota

**Fig 4:**
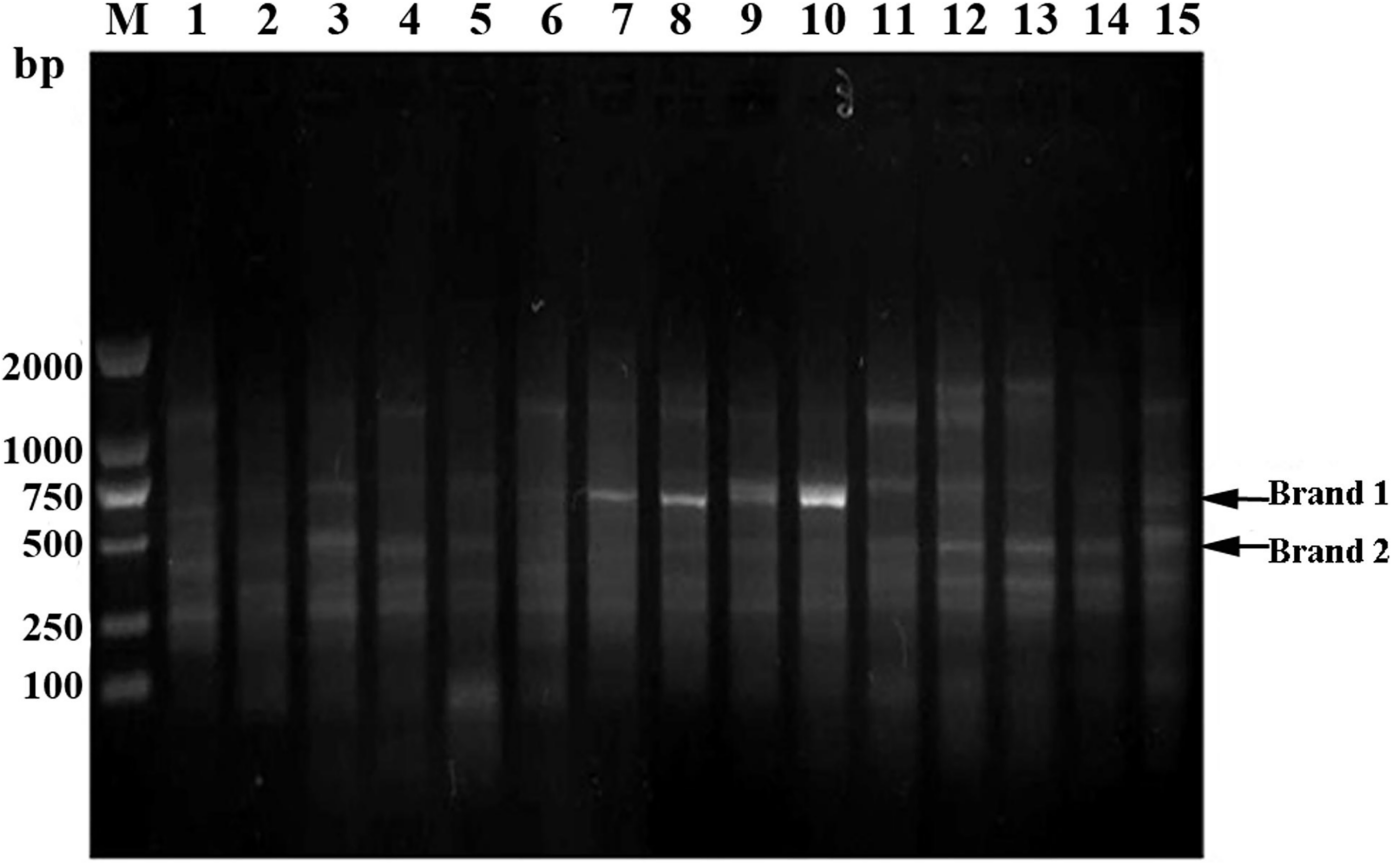
ERIC-PCR fingerprint analysis of gut microbiota. ERIC-PCR analysis was used to analyze the microbial community structure of rats. The results indicated significant alterations in the gut microbiota of rats in the alcohol model group, with an obvious brand at 750 bp, which was not clear in the control and aplysin-treatment groups. M: DL2000 DNA Marker; 1–5: control group; 6–10: alcohol model group; 11–15: aplysin intervention group.

ERIC is one repetitive sequence of genes that mainly exists in intestinal bacteria and has a length of 127 bp. There is specificity for the distribution of the sequence in the intestinal bacterial chromosome and its copy number. Therefore, ERIC-PCR analysis was used to analyze the microbial community structure of rats. The results of ERIC-PCR indicated significant alterations in the gut microbiota of rats in the alcohol model group, with an obvious band at 750 bp, which was not clear in the control and aplysin-treatment groups (Fig 4).

### 16S rDNA high throughput sequencing analysis

**Fig 5:**
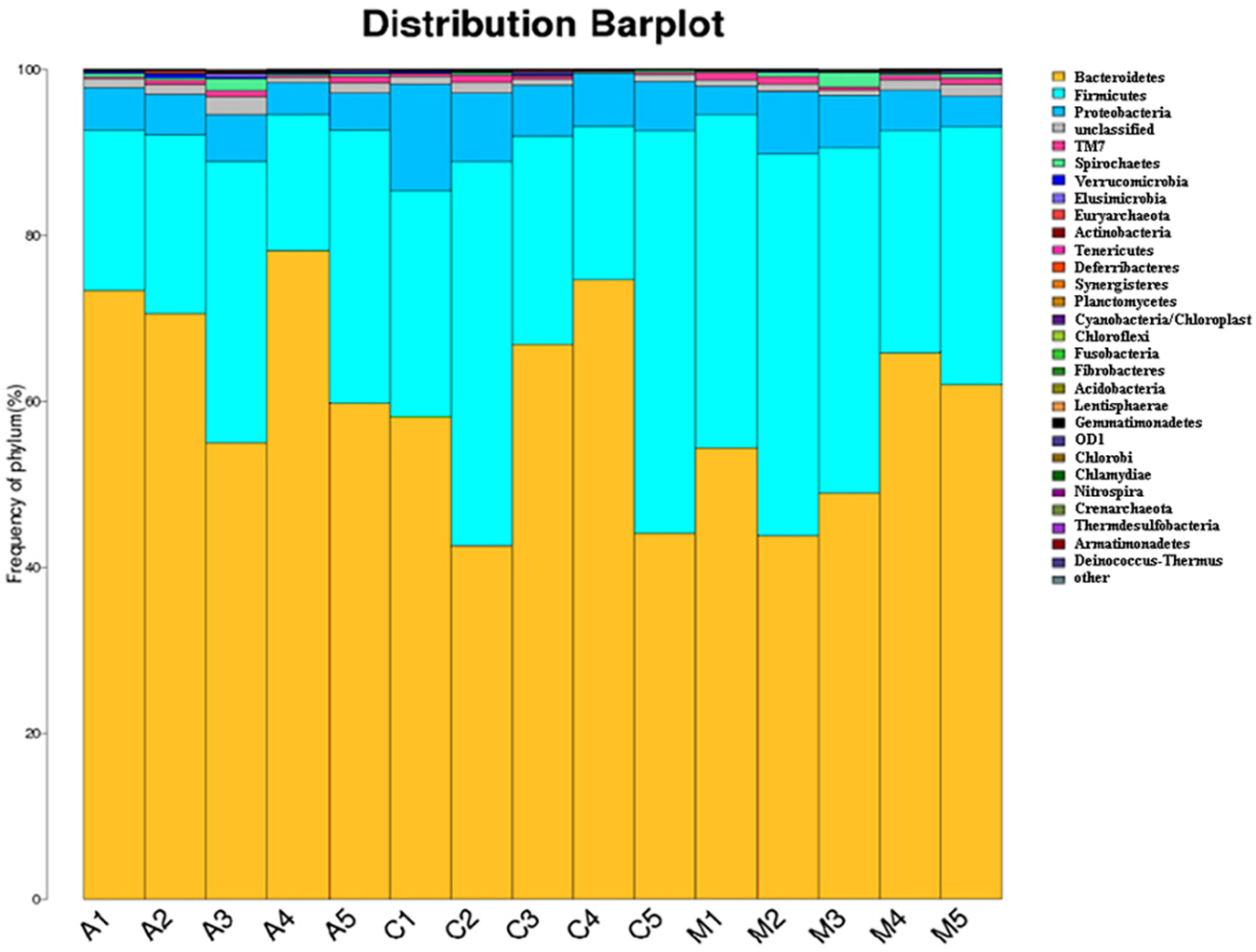
The bacterial composition of all the samples at the phylum level. **]** At the phylum level, bacteria associated with 29 strains of nematodes were detected by 16S rDNA high throughput sequencing analysis. Bacteroidetes was the most predominant phylum, followed by Firmicutes and Proteobacteria. Each color represents one type of bacterial strain, and the area of the histogram of each color represents the number ratio of bacteria strains. C: control group; M: alcohol model group; A: aplysin intervention group.

Then, 16S rDNA high throughput sequencing was used for the analysis of the molecular ecology of fecal flora. An average of 4726 OTUs per sample were determined, with a maximum of 4972 and a minimum of 3333 identified OTUs. The OTUs of bacteria associated with nematodes were classified and organized. At the phylum level, bacteria associated with 29 strains of nematodes were detected. Fig 5 shows the bacterial composition of all samples at the phylum level. The most abundant phyla included Bacteroidetes, Firmicutes and Proteobacteria.

**Fig 6:**
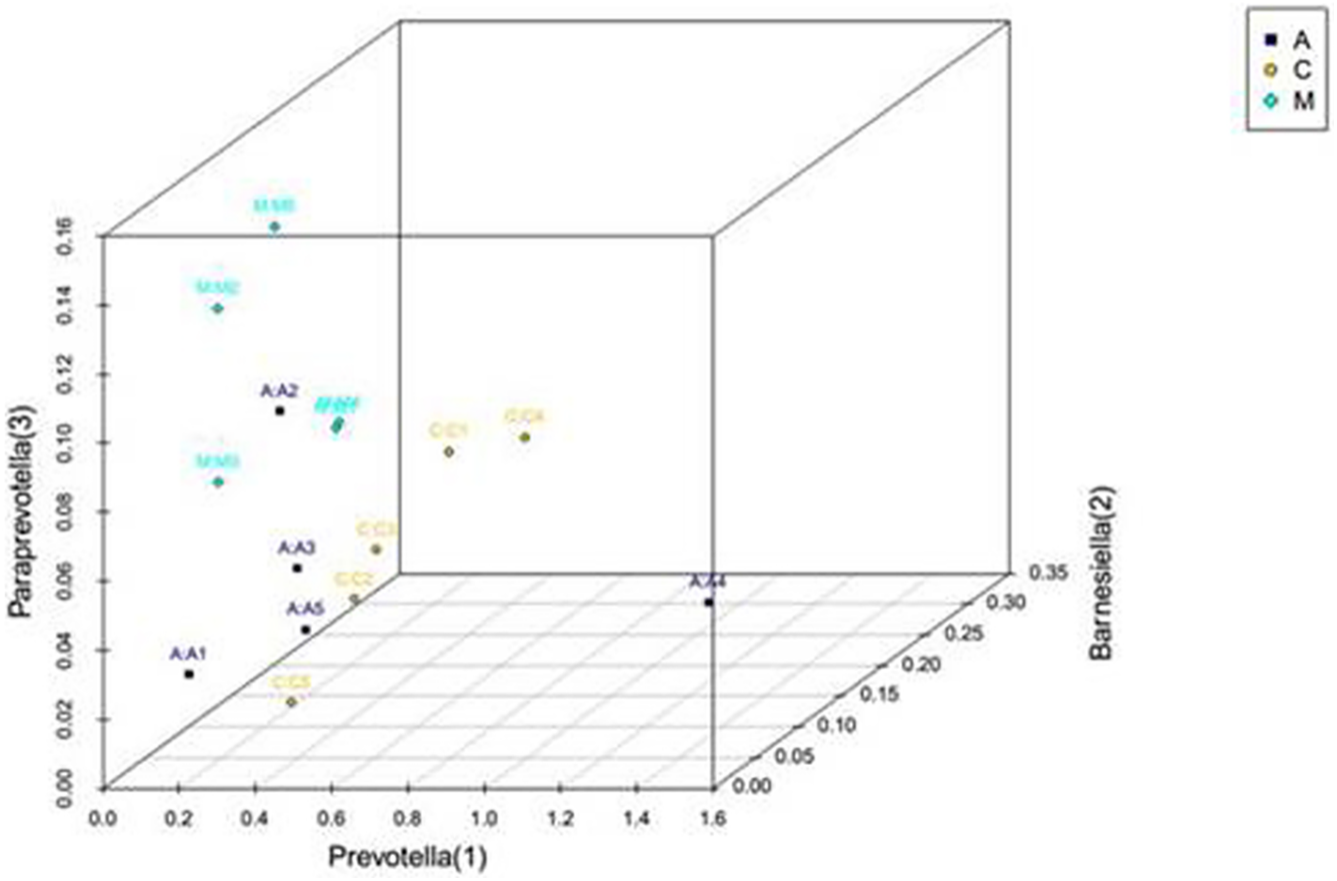
Three-dimensional figure of the first three RDP distributions. PCoA (principal co-ordinates analysis) plot based on OTU abundance. Each point represents the fecal microbiota of rat. Samples with higher similarity in the diagram were gathered, whereas samples with lower similarity are farther in distance. C: control group; M: alcohol model group; A: aplysin intervention group.

At the phylum level, bacteria associated with 29 strains of nematodes were classified into 120~190 taxonomic genus in different groups. At the genus level, alcohol feeding for 8 weeks resulted in obvious changes in the gut microbial community. The relative abundances in the top three genera were Prevotella, Barnesiella and Paraprevotella, respectively, which were all from the Bacteroidetes phylum. As shown in Fig 6, the abundance of Paraprevotella in the alcohol model group was higher than that in the control group. Aplysin treatment reduced the abundance of Paraprevotella (*P*< 0.05). The abundance of Prevotella in the alcohol model group was decreased, but there was no statistically significant difference compared to that in the control group. However, the abundance of Prevotella in the aplysin group was higher than that in the alcohol model group (*P*< 0.05).

**Fig 7:**
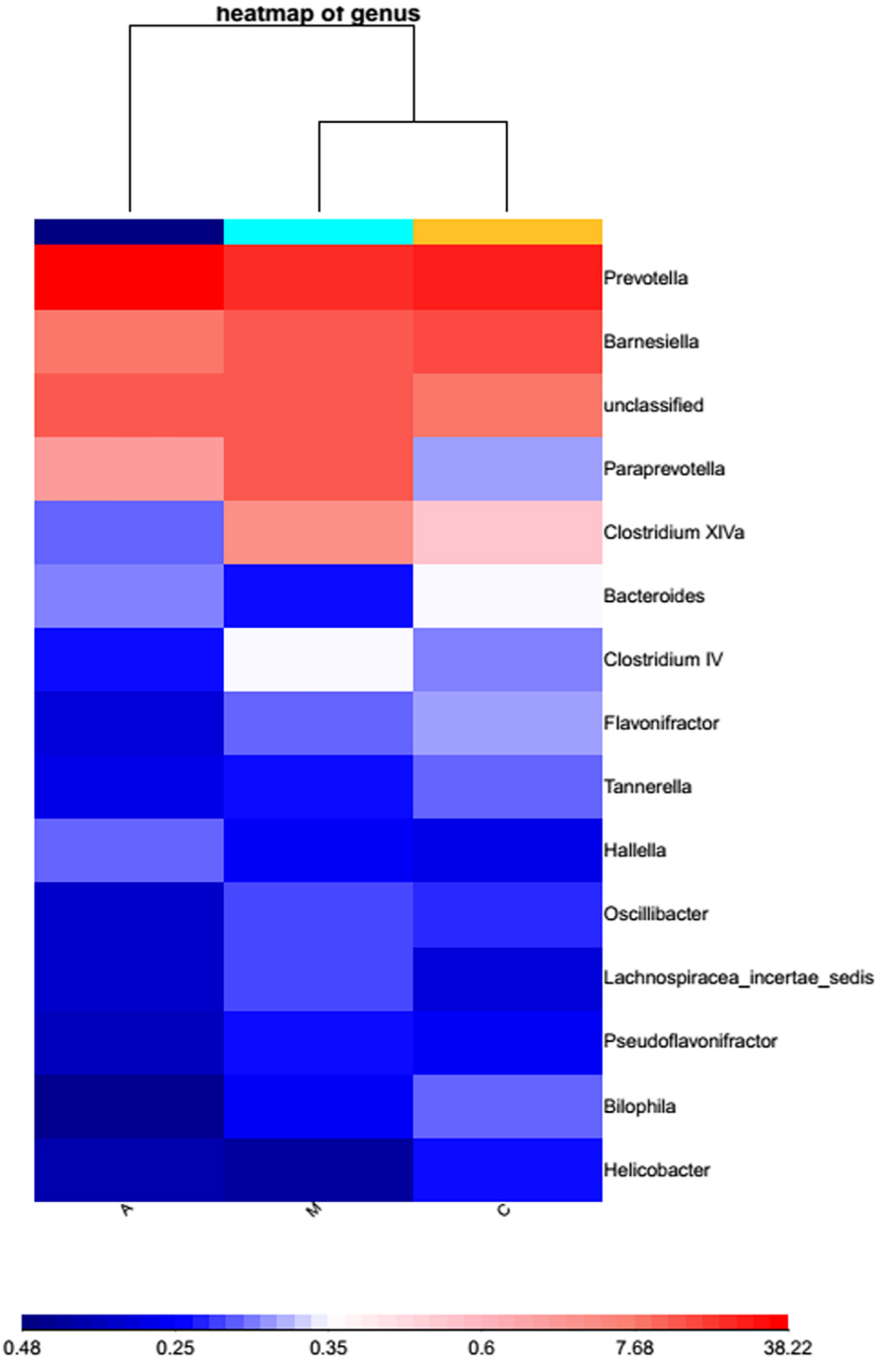
Heat map of the top 15 genera in different groups. The genus abundance heat map was made with a species abundance matrix. Each column represents a sample, and each line represents the bacterial genus. The color block represents the relative abundance. Higher relative abundance was indicated by the colorred, and lower abundance was indicated by the color blue. C: control group; M: alcohol model group; A: aplysin intervention group.

Among all genera, whose abundance ranked 4th ~ 14th, alcohol feeding deceased the abundances of Bacteroides from the Bacteroidetes phylum and Helicobacter from the Proteobacteria phylum and increased the abundance of Clostridium IV from the Firmicutes phylum compared with those in the control group (*P*< 0.05). The abundance of Clostridium XlVa from the Firmicutes phylum in the alcohol model group was increased, but there was no statistically significant difference compared to that in the control group. Aplysin treatment reduced the abundance of Clostridium IV and Clostridium XlVa from the Firmicutes phylum compared to those in the alcohol model group (*P*< 0.05, Fig 7).

In addition, alcohol feeding resulted in changes in other bacterial abundances at the genus level, with increased abundances of Meniscus, Anaerophaga and Rikenella all from the Bacteroidetes phylum, Anaerostipes and Peptococcus from the Firmicutes phylum, and Treponema from the Spirochaetes phylum. The abundances of Blautia, Lactobacillus and Schwartzia from the Firmicutes phylum and Delftia and Gemmiger from the Proteobacteria phylum decreased compared with those in the control group (*P*< 0.05). However, aplysin treatment reduced the abundance of Anaerophaga, Anaerostipes and Peptococcus compared with those in the alcohol model group (*P*< 0.05, Table 2).

**Table 2:** The intestinal bacteria distribution of the representative bacterial species ($\bar{x}$ ± S).

phylum	family	genus	abundance(%)		
			Control	Model	Aplysin
Bacteroidetes	Bacteroidaceae	Bacteroides	4.63±1.54	2.16±0.74*	3.02±1.48
	Cytophagaceae	Meniscus	0.28±0.21	0.75±0.43*	0.68±0.18
	Marinilabiaceae	Anaerophaga	0.63±0.31	1.37±0.36*	0.85±0.11^**#**^
	Prevotellaceae	Paraprevotella	3.20±1.87	7.68±2.29*	4.91±0.94^**#**^
		Prevotella	31.35±7.53	21.89±6.46	38.22±11.06^**#**^
	Rikenellaceae	Rikenella	0.56±0.35	1.30±0.64*	0.99±0.26
Firmicutes	Lachnospiraceae	Blautia	0.11±0.08	0.02±0.01*	0.03±0.03
		Anaerostipes	0.02±0.01	0.13±0.09*	0.01±0.01^**#**^
		Clostridium XlVa	3.69±1.80	6.85±4.28	2.84±1.33^**#**^
	Lactobacillaceae	Lactobacillus	1.16±0.93	0.13±0.13*	0.11±0.11
	Peptococcaceae	Peptococcus	0.06±0.04	0.13±0.06*	0.05±0.01^**#**^
	Ruminococcaceae	Clostridium IV	2.17±1.27	3.87±1.06*	2.06±0.45^**#**^
	Veillonellaceae	Schwartzia	2.26±1.23	0.84±0.67*	0.45±0.19
Proteobacteria	Comamonadaceae	Delftia	0.75±0.19	0.30±0.14*	0.42±0.26
	Helicobacteraceae	Helicobacter	2.49±0.60	0.64±0.30*	0.95±0.39
	Hyphomicrobiaceae	Gemmiger	0.13±0.05	0.06±0.04*	0.05±0.03
Spirochaetes	Spirochaetaceae	Treponema	0.12±0.08	0.33±0.16*	0.26±0.10

Alcohol feeding resulted in changes in many bacteria abundances at the genus with increased abundances of Meniscus, Anaerophaga and Rikenella all from the Bacteroidetes phylum, Anaerostipes and Peptococcus from the Firmicutes phylum, and Treponema from the Spirochaetes phylum. The abundances of Blautia, Lactobacillus and Schwartzia from the Firmicutes phylum, and Delftia and Gemmiger from the Proteobacteria phylum decreased, compared with those in the control group. However, aplysin treatment reduced the abundance of Anaerophaga, Anaerostipes and Peptococcus, compared with those in the alcohol model group.

*P< 0.05 *vs* Control

^#^*P*< 0.05 *vs* Model.

### The correlation analysis between gut microbiota and serological indexes

Bivariate borrelations analysis was performed between the 17 genera that were changed by alcohol or aplysin intervention in all the groups of rats. Anaerophaga and Anaerostipes were both positively correlated with ALT and ALP. Prevotella was negatively correlated with ALP and FABP2. Clostridium IV was significantly positively correlated with ALP. Peptococcus was positively correlated with FABP2, but negatively correlated with IL-10 (Tables 3–5).

**Table 3:** Correlation analysis between gut microbiota composition and enzymology indexes of liver function.

genus	ALT		AST		ALP	
	r	P	r	P	r	P
Bacteroides	-0.28	0.35	-0.09	0.78	0.09	0.75
Meniscus	0.23	0.44	0.52	0.07	0.07	0.83
Anaerophaga	**0.62**	**0.03**	0.32	0.28	**0.57**	**0.04**
Paraprevotella	0.14	0.64	0.09	0.76	0.26	0.38
Prevotella	-0.19	0.54	-0.06	0.83	**-0.61**	**0.03**
Rikenella	0.11	0.71	-0.54	0.06	0.29	0.34
Blautia	-0.40	0.18	-0.34	0.26	-0.30	0.32
Anaerostipes	**0.56**	**0.04**	0.01	0.99	**0.56**	**0.04**
Clostridium XlVa	-0.06	0.83	-0.14	0.65	0.15	0.62
Lactobacillus	-0.38	0.21	-0.16	0.61	-0.32	0.28
Peptococcus	0.34	0.26	0.02	0.95	0.47	0.11
Clostridium IV	0.51	0.08	0.15	0.64	**0.56**	**0.04**
Schwartzia	-0.15	0.62	-0.20	0.51	0.12	0.70
Delftia	0.26	0.40	0.24	0.42	0.38	0.20
Helicobacter	-0.21	0.49	0.24	0.43	-0.15	0.96
Gemmiger	-0.32	0.29	-0.22	0.47	0.17	0.58
Treponema	-0.05	0.86	0.24	0.44	-0.29	0.34

**Table 4:** Correlation analysis between gut microbiota composition and the levels of plasma inflammatory factor.

genus	TNF-α		TGF-β		IL-10	
	r	P	r	P	r	P
Bacteroides	0.04	0.91	-0.34	0.25	0.36	0.23
Meniscus	0.29	0.34	-0.14	0.64	-0.13	0.66
Anaerophaga	-0.01	0.97	0.11	0.73	-0.16	0.60
Paraprevotella	-0.12	0.69	0.17	0.58	0.24	0.43
Prevotella	0.01	0.99	0.15	0.63	0.34	0.25
Rikenella	0.04	0.89	0.05	0.87	0.28	0.36
Blautia	0.24	0.42	0.01	0.98	0.25	0.40
Anaerostipes	-0.03	0.93	-0.01	0.97	-0.31	0.30
Clostridium XlVa	-0.06	0.86	-0.21	0.49	0.21	0.49
Lactobacillus	0.42	0.15	0.05	0.86	-0.04	0.89
Peptococcus	0.06	0.85	023	0.46	**-0.67**	**0.01**
Clostridium IV	0.04	0.90	-0.04	0.90	-0.40	0.17
Schwartzia	-0.23	0.46	-0.03	0.93	-0.26	0.39
Delftia	-0.13	0.68	-0.19	0.53	0.39	0.18
Helicobacter	-0.01	0.99	-0.26	0.39	0.04	0.89
Gemmiger	0.06	0.84	-0.04	0.91	-0.37	0.22
Treponema	-0.13	0.67	0.03	0.92	-0.27	0.37

**Table 5:** Correlation analysis between gut microbiota composition and serum indexes of intestinal mucosal barrier function.

genus	endotoxin		DAO		DLA		FABP2	
	r	P	r	P	r	P	r	P
Bacteroides	-0.15	0.62	-0.02	0.96	0.08	0.80	-0.20	0.53
Meniscus	0.22	0.47	-0.18	0.53	0.27	0.34	0.16	0.63
Anaerophaga	0.07	0.83	0.01	0.99	0.28	0.33	0.37	0.23
Paraprevotella	0.14	0.64	0.13	0.67	-0.10	0.74	0.03	0.94
Prevotella	-0.16	0.60	0.38	0.19	0.19	0.52	**-0.66**	**0.02**
Rikenella	0.48	0.09	0.25	0.40	0.01	0.99	-0.55	0.07
Blautia	0.19	0.50	-0.24	0.42	-0.24	0.41	-0.27	0.40
Anaerostipes	0.31	0.29	-0.03	0.91	0.09	0.76	0.46	0.14
Clostridium XlVa	-0.13	0.64	-0.27	0.36	-0.16	0.59	0.01	0.97
Lactobacillus	-0.32	0.28	-0.29	0.31	-0.22	0.45	0.04	0.90
Peptococcus	0.49	0.09	-0.01	0.96	0.13	0.66	**0.74**	**0.01**
Clostridium IV	0.18	0.55	-0.18	0.53	0.11	0.70	0.58	0.06
Schwartzia	-0.45	0.12	-0.08	0.79	-0.23	0.42	0.36	0.25
Delftia	-0.16	0.59	-0.05	0.86	0.08	0.79	0.10	0.77
Helicobacter	-0.36	0.23	-0.27	0.35	0.15	0.62	0.24	0.46
Gemmiger	0.47	0.12	0.33	0.25	-0.20	0.50	0.51	0.09
Treponema	0.32	0.29	0.14	0.63	-0.12	0.68	0.05	0.87

Bivariate correlations analysis was performed between the 17 genera that were changed by alcohol or aplysin intervention in all the groups of rats. Anaerophaga and Anaerostipes were both positively correlated with ALT and ALP. Prevotella was negatively correlated with ALP and FABP2. Clostridium IV was significantly positively correlated with ALP. Peptococcus was positively correlated with FABP2, but negatively correlated with IL-10. Significant correlations (*P*< 0.05) are in bold.

## Discussion

It was reported recently that ethanol-induced dysbiosis can disrupt the integrity of the intestinal epithelium, resulting in intestinal inflammation and bacterial translocation [31]. Gut-derived bacterial endotoxins induce a hepatic necroinflammatory cascade and reduce hepatic damage in ALD. The current study, for the first time, highlights the protective effect of aplysin, a brominated sesquiterpene, on the gut microbiota and the intestinal mucosal barrier against ethanol-induced liver injury in rats.

Our previous studies have demonstrated that a sequential increase in the alcohol dose (8~12 mL kg^-1^ bw) induces hepatic injury and overcomes tolerance caused by the alcohol consumption at the same dose [15, 20]. The present study showed that enzyme activities of serum AST, ALT and ALP and hepatic TG level in the alcohol model group were significantly increased compared with those in the control group after 8 weeks of alcohol feeding. There were hepatic histopathological and ultrastructure changes in alcohol-fed rats, including lipid droplet accumulation, inflammatory cell infiltration, microvesicular steatosis, swelling mitochondria and Mallory body. These findings confirm that chronic alcohol feeding leads to liver damage. However, aplysin intervention reduced levels of serum AST, ALT and ALP and hepatic TG at the end of the study, and alleviated alcohol-induced fat accumulation, mitochondria swelling and inflammation in the liver.

The current data suggest that alcohol administration elevates levels of TNF-α and TGF-β, and upregulates the inflammatory response. At the same time, it reduces the level of anti-inflammatory IL-10. These findings are consistent with previous reports [11, 32]. Chronic alcohol exposure plays an important role in initiating and promoting alcohol-induced hepatic damage. It could activate the innate immune and product proinflammatory cytokines, including TNF-α and TGF-β. These alcohol-induced effects could be reversed by aplysin treatment. IL-1β and TNF-α levels in the aplysin group were decreased, and the level of IL-10 was increased. Louis et al. [33] found that IL-10 possessed a hepatic protective effect on proliferation and fibrosis. IL-10 could also reduce the production of proinflammatory cytokines, such as TNF-α and IL-1β, activate signal transducers and activators of transcription 3 (STAT3) in Kupffer cells and subsequently inhibit liver inflammation [34]. Our results show that aplysin can normalize pro- and anti-inflammatory cytokine secretions in alcohol-fed rats.

Moreover, increased gut permeability and intestinal bacterial endotoxin can trigger an immune response and produce proinflammatory cytokines, such as IL-1, IL- 6 and TNF-α, and induce hepatic injury [35]. Endotoxemia is closely related to the pathogenesis of ALD and stimulates the production of proinflammatory cytokines [36]. This study suggests that alcohol feeding increases serum endotoxin level. It was reported that several hypotheses are involved in the pathogenesis of endotoxemia, such as bacterial overgrowth in the intestine and increased gut permeability. Therefore, the intestinal mucosal barrier function was determined in this study. Chronic ethanol feeding led to histological changes in the small intestinal structure. The structure of small intestinal villi showed obvious atrophy, sparse arrangement, and enlarged tight connections. Serological indexes detection revealed that the levels of plasma DAO, D-LA and FABP2 in the alcohol model group were increased. The intact intestinal mucosa provides a barrier function to prevent DAO and D-LA from infiltrating the portal circulation, which are indexes of increases in permeability of the intestinal wall [37]. In this study, after aplysin intervention, the small intestine columnar epithelium and microvilli arrangement were improved, the swelling of tight connections was reduced, and the levels of plasma DAO and FABP2 were reduced. Consecutively, the level of plasma endotoxin was almost back to normal. The data revealed that aplysin had a protective effect on the intestinal mucosal barrier and maintained intestinal permeability in rats with alcohol exposure. Endotoxemia was therefore prevented.

Alcohol intake can alter the intestinal microbiota and cause changes in the number and proportion of bacteria in the small intestine [4]. These changes are relative to ecological imbalance, and the gut microbiota can sense signals of disruption of the intestinal mucosal cells; the transfer and response of the intestinal microbiota can cause inflammatory disorders. Furthermore, intestinal dysbacteriosis caused the mucosa to lack the necessary micronutrients (e.g., short chain fatty acids, etc.) and oxidation reduction potential. This also caused the intestinal permeability to increase and damaged the intestinal mucosal barrier [38]. Therefore, the gut microbiota in rats with ethanol-induced liver injury was detected in the present study. The results of ERIC-PCR indicated significant alterations in the gut microbiota of alcohol-fed rats, with an obvious band at 750 bp. Aplysin treatment shifted the overall structure of the ethanol-disrupted gut microbiota toward that of the control group. Furthermore, in each group, 120 to 190 genera of bacteria were detected by 16s rDNA high throughput sequencing. Ethanol induced widespread changes in gut microbial composition, with increased abundances of Paraprevotella, Clostridium IV, Meniscus, Anaerophaga, Rikenella, Anaerostipes and Peptococcus and decreased abundances of Bacteroides, Helicobacter, Blautia, Lactobacillus, and Schwartzia. However, aplysin intervention decreased abundances of Paraprevotella, Clostridium IV, Clostridium XlVa, Anaerophaga, Anaerostipes and Peptococcus and increased the abundance of Prevotella.

It is worth noting that chronic ethanol feeding increased the abundance of Paraprevotella, which was reduced after aplysin intervention. Paraprevotella is a type of bacterial genus that produces succinate [39], which is a citric acid cycle intermediate. This indicates the relationship of Paraprevotella and its metabolites produce succinate during an alcohol-induced liver injury and the protective effect of aplysin. Chouchani et al. [40] found that selective accumulation of succinate is a universal metabolic signature of ischemia in a range of tissues and is responsible for mitochondrial reactive oxygen species (ROS) production, which initiates oxidative damage, cell death and aberrant immune responses. There are many studies that suggest that oxidative stress plays an important role in the development of alcoholic liver disease [41]. Our study showed that aplysin treatment reduces the abundance of Paraprevotella. It also demonstrated that the adjustment to gut bacteria might be a mechanism in the protection of aplysin in alcoholic liver injury.

Bacteroides could be the most powerful inducer of regulatory T cells and stimulate the production of the anti-inflammatory cytokine IL10 by intestinal macrophages [42]. Lactobacillus can ferment carbohydrates to produce lactic acid and has many positive physiological effects. It can prevent harmful bacteria from adhering to the intestinal epithelial, enhance the barrier function of intestinal epithelial cells, stimulate the production of immunoglobulin, and enhance the host immune system [43]. Our data showed that the abundances of Bacteroides and Lactobacillus were decreased by alcohol intake.

Prevotella is a type of opportunistic pathogen. In vitro studies have demonstrated that lipopolysaccharides (LPS) on the surface of Prevotella can significantly promote the production of IL-8 by human fibroblasts, strengthening the inflammatory response [44]. However, in this study, Prevotella was negatively correlated with ALP and FABP2. The results showed the protection of Prevotella in alcoholic liver injury in the intestinal mucosal barrier, and aplysin treatment increased the abundance of Prevotella.

Clostridium IV, Clostridium XlVa, and Anaerostipes can produce butyrate and are in intestinal probiotics [45]. However, the abundances of these species increased after suffering alcohol damage in this study. Zhang et al. [46] found that Anaerostipes significantly aggravated colitis in dextran sulfate sodium (DSS)-treated mice while exerting no detrimental effect in healthy mice. Our study also showed that Clostridium IV was significantly positively correlated with ALP, and Anaerostipes was positively correlated with ALT and ALP. Further experiments should be performed to confirm whether these bacterial strains play a negative role in alcoholic liver injury.

Anaerophaga is a moderately thermophilic, strictly anaerobic bacterium with xylanase activity [47]. Anaerophaga was positively correlated with ALT and ALP. The anaerobic bacterium, Peptococcus, is a type of opportunistic pathogen and considered an important pathogen in the etiology of mixed anaerobic infections [48]. Peptococcus was positively correlated with FABP2 but negatively correlated with IL-10. The abundances of Anaerophaga and Peptococcus were increased in ethanol-fed rats but decreased after aplysin intervention.

The mechanisms between microbiota composition and maintenance of the intestinal mucosal barrier are diverse and complicated. In many cases, it is usually not a single strain that plays a decisive role but the gut microbiota composition or the proportion of bacterial species. These bacterial strains involved in alcohol-induced liver injury and the protective effect of aplysin need to be further studied to explore their potential relationship.

In conclusion, this study indicated that chronic alcohol consumption could induce a wide range of changes in the gut microbiota, damage the intestinal mucosal barrier, result in endotoxemia and influence the inflammatory condition of rats. Aplysin exerted a protective effect on ethanol-induced hepatic injury, normalized fecal microbiota composition, repaired intestinal barrier function and thus provided a low plasma endotoxin level and inflammatory response. These new findings suggest that aplysin can be regarded as a gut microbiota-targeted strategy to combat ethanol-induced liver injury.

## Supporting information

S1 File Raw sequence reads of fecal flora. The categories, sequences and abundance of the bacteria that were detected in the samples were listed in the table.
(XLS)
